# Beyond writing machines: A Kano model analysis of researchers’ hierarchical needs for AIGC services across the research lifecycle

**DOI:** 10.1371/journal.pone.0344849

**Published:** 2026-03-12

**Authors:** Yong Kong, Tongqiang Dong, Ronglong Chen, Yunming Wu, Ziyi Yang

**Affiliations:** Faculty of education, Qufu Normal University, Rizhao, China; Philadelphia University, JORDAN

## Abstract

The proliferation of AI-Generated Content (AIGC) tools presents both opportunities and challenges for the academic service ecosystem. However, a systematic understanding of researchers’ multifaceted demands for AIGC functionalities remains underdeveloped, hindering the strategic design and optimization of these services. This study addresses this gap by investigating three core questions: (1) What specific AIGC service functions do researchers desire across the research lifecycle? (2) How can these needs be categorized hierarchically? (3) What is their relative importance in influencing user satisfaction? Employing an exploratory sequential mixed-methods design, this research first identified a comprehensive list of 15 service demands through semi-structured interviews with 45 expert researchers (N = 45). Subsequently, these demands were prioritized through a large-scale questionnaire survey involving 412 researchers (N = 412), utilizing the Kano model and Importance-Performance Analysis. The results reveal a clear hierarchy of needs: we identified three must-be attributes (e.g., data security, citation accuracy), seven one-dimensional attributes (e.g., automated literature summarization, language polishing), and five attractive attributes (e.g., generating novel research hypotheses, smart journal recommendation). These findings provide a detailed framework for AIGC service development and offer an evidence-based model for academic institutions to prioritize resource allocation, thereby enhancing the value and adoption of AIGC in scholarly research.

## Introduction

With the rapid proliferation of Artificial Intelligence Generated Content (AIGC) models, the issue of general accessibility to advanced language tools has been largely resolved, providing researchers with powerful new capabilities [1,2]. However, the problem of service applicability—the precise alignment of AIGC functions with the complex, multi-stage demands of the academic research lifecycle—remains inadequately addressed [3]. Service applicability emphasizes the personalization and contextualization of tools to meet the differentiated needs of researchers across diverse fields and career stages. Current AIGC services often focus on isolated tasks like text generation or summarization [4], but this development frequently proceeds without a deep, systematic understanding of researchers’ actual, hierarchical needs. Consequently, most research on AIGC in academia centers on technical benchmarks or broad ethical discussions [5], often overlooking the granular, workflow-integrated demands of the researchers themselves. This phenomenon results in a disconnect where AIGC tools, despite their power, are not optimally integrated into the nuanced process of scientific inquiry.

To bridge this gap and enhance the applicability of AIGC for academia, it is necessary to adopt a demand-driven innovation model, a concept increasingly relevant in technology design [6]. Originating in economics and management, this philosophy emphasizes that user needs should be the primary catalyst for product and industrial innovation [7]. Applying this to AIGC services involves creating an adaptive feedback loop between service features and the evolving requirements of researchers [8]. This approach moves beyond simply providing a “writing machine” and aims to develop a holistic support system that intelligently assists researchers throughout their entire lifecycle [9]. Therefore, the central objective of this study is to develop a hierarchical demand framework for AIGC services in academia by systematically identifying, classifying, and prioritizing researchers’ needs. To achieve this, we address the following research questions:

RQ1: What specific AIGC service functions do researchers desire across the entire research lifecycle?

RQ2: How can these needs be classified into a hierarchical structure of Must-be, One-dimensional, and Attractive qualities?

RQ3: What is the relative importance of these needs categories in driving user satisfaction and informing strategic service design?

This study makes three primary contributions. Theoretically, it moves beyond generic acceptance models to provide a granular, three-tier hierarchical needs framework tailored to the academic domain. Methodologically, it demonstrates the efficacy of integrating the Kano model with computational thematic analysis as a robust approach for user-centered design in the context of emerging AI technologies [10,11]. Practically, the findings provide a data-driven blueprint for developers to design more valuable AIGC services and for academic institutions to prioritize resource allocation [12], ultimately accelerating scientific discovery and enhancing research productivity.

**Fig 1:**
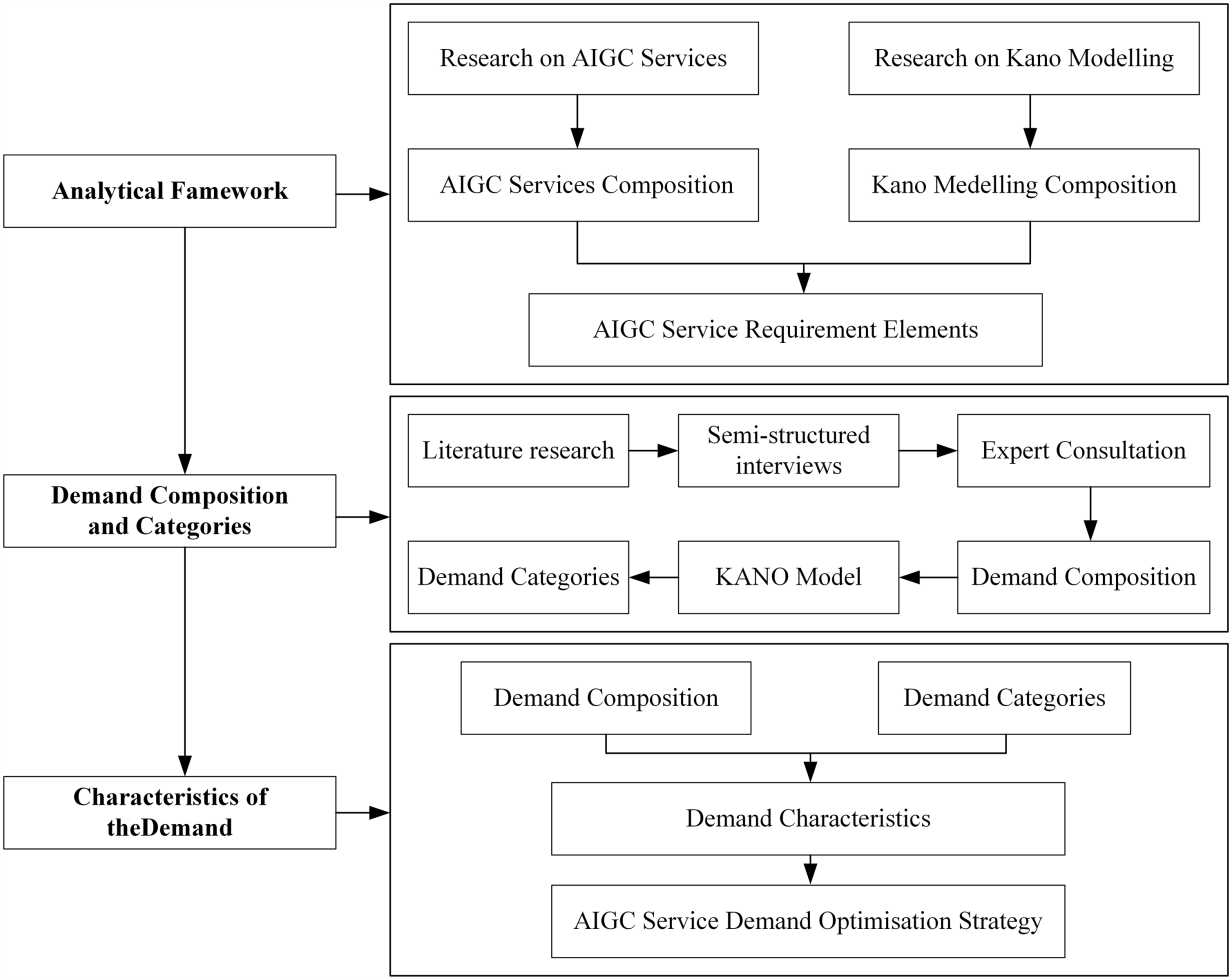
Research process.

This study distinguishes itself from previous technology-centric research by systematically analyzing AIGC service requirements from a user-demands perspective. Our research proceeds by first establishing an analytical framework that maps AIGC services to the research lifecycle (Fig 1). Guided by this framework, we employ a sequential mixed-methods approach: we initially identify core AIGC service demands through qualitative interviews and computational thematic analysis using BERTopic [13], then use the Kano model to quantitatively classify these demands into a needs hierarchy. Finally, based on these findings, we analyze the overarching characteristics of researcher needs and formulate strategic recommendations for the development of next-generation AIGC services.

## Literature review

### The emergence and application of AIGC in the scholarly ecosystem

The integration of Artificial Intelligence Generated Content (AIGC) into the scholarly ecosystem represents a paradigm shift in knowledge production [14], moving from early speculative discourse on ethical quandaries [15,16] to a rapidly expanding body of empirical research. Current studies confirm AIGC’s functional utility across various academic tasks. The most prominent research stream concentrates on its role in academic writing and literature synthesis, demonstrating efficacy in drafting manuscripts, enhancing language clarity, and generating preliminary summaries [17]. Another significant area is AIGC’s utility in data analysis and coding, where it functions as a co-pilot for generating programming scripts and interpreting statistical outputs [18,19].

Recent survey data underscores this rapid adoption, with a significant portion of researchers already incorporating these tools into their workflows, albeit with notable disciplinary variations [20]. Theoretically, this adoption can be partially explained by the Task-Technology Fit (TTF) model, which posits that technology is more likely to be used if its capabilities match the tasks users must perform [21]. For researchers, AIGC tools that effectively reduce the cognitive load of routine tasks like formatting or summarizing literature exhibit a strong TTF [22]. However, while these studies validate AIGC’s utility for discrete tasks, they collectively conceptualize it as a collection of task-specific assistants rather than an integrated support system [23], leaving its potential for holistic workflow integration largely unexplored.

### Critical gaps in current research: Superficial needs analysis and fragmented perspectives

Despite the rapid accumulation of research, two critical limitations impede a deeper understanding of how to strategically design AIGC services for academia.

First, the dominant theoretical lens for understanding user-AIGC interaction has been technology acceptance models (e.g., TAM, UTAUT) [24,25]. While instrumental in predicting usage intention based on constructs like perceived usefulness [26,27], these models treat user needs as a monolithic, linear construct. They ascertain if a service is useful but fail to differentiate the heterogeneous nature and hierarchical structure of user requirements. This linear view creates a ‘utility paradox,’ where high adoption rates can mask low user satisfaction and suboptimal integration into complex scholarly workflows.For instance, they cannot distinguish a foundational ‘must-be’ feature whose absence causes extreme dissatisfaction (e.g., data privacy) from an innovative ‘attractive’ feature whose presence creates unexpected delight (e.g., novel hypothesis generation) [28]. This leads to a superficial understanding of what truly drives user satisfaction and loyalty.

Second, existing studies are characterized by a fragmented, task-oriented perspective. The focus on isolated functions like “writing” or “summarizing” overlooks that scholarly research is a complex, interconnected lifecycle—a journey from ideation through to publication and dissemination [29]. Analyzing user needs within siloed tasks risks misinterpreting their relative importance and failing to identify needs that arise from the interplay between different stages. This fragmentation is not merely a methodological oversight; it creates a significant blind spot, preventing a holistic understanding of how AIGC can be systemically integrated to support the entire scholarly workflow [30]. Moreover, it sidesteps crucial debates around “cognitive offloading,” where over-reliance on task-specific tools may inadvertently hinder the development of researchers’ core critical thinking skills [31,32].

Furthermore, while established models like TAM and TTF provide a foundational understanding of technology adoption, they often frame the user as a passive recipient of a static tool. This perspective is insufficient for capturing the dynamic and interactive nature of AIGC in research. The relationship is not merely about ‘adopting’ a finished product, but about engaging in a co-evolutionary partnership where both the researcher’s workflow and the AI’s utility evolve through continuous interaction.

Therefore, a more fitting theoretical lens is Human-Centered AI (HCAI), as advocated by Albekairi [8]. HCAI emphasizes designing AI systems that serve as powerful partners, amplifying human creativity and control rather than replacing human judgment. This framework shifts the focus from simple ‘usefulness’ and ‘ease of use’ to more nuanced goals like empowerment, augmentation, and trustworthy collaboration. Our study aligns with this perspective by investigating how AIGC can be designed not just to be adopted, but to become an integral and reliable partner in the scholarly process.

### A new theoretical lens: The Kano model for prioritizing needs in emerging technologies

To overcome these limitations, this study adopts the Kano model as its core theoretical framework. This model is uniquely suited for our research objective because it moves beyond simple acceptance to provide a nuanced understanding of the asymmetric and non-linear relationship between service attributes and user satisfaction—a critical factor in the context of a disruptive technology like AIGC.

The Kano model posits that user requirements are not homogenous but can be classified into three primary categories based on their impact on satisfaction: Must-be attributes, whose absence causes extreme dissatisfaction but whose presence is taken for granted (e.g., a hotel room must be clean); One-dimensional (or Performance) attributes, where satisfaction is directly proportional to the level of fulfillment (e.g., higher internet speed); and Attractive attributes, which are unexpected and create delight when present but cause no dissatisfaction when absent (e.g., a complimentary room upgrade) [10].

The model’s primary strength, and the core reason for its selection, lies in its ability to deconstruct the generic concept of “usefulness” employed by traditional acceptance models like TAM. While TAM can predict if a technology will be used, the Kano model explains how specific features contribute to satisfaction and loyalty, which is essential for strategic design [11]. In the rapidly evolving AIGC domain, where user expectations are still being formed, this distinction is paramount. For instance, data security is a Must-be attribute; its failure would destroy trust, regardless of other powerful features. Conversely, the ability to generate novel research hypotheses is an Attractive attribute that can create significant competitive advantage and user delight. Linear models like TAM are incapable of capturing this crucial hierarchical differentiation [33].

Furthermore, the Kano model is especially powerful when applied to emerging technologies because it helps identify latent and unarticulated needs, which are often the source of Attractive, innovative features. Users may not be able to express a desire for a function they have never imagined. This necessitates a mixed-methods approach, which we adopt in this study. The initial qualitative phase (interviews and thematic analysis) is essential for discovering the full range of potential service demands—both explicit and latent. The subsequent quantitative phase (the Kano questionnaire) is then used to classify these discovered demands into the hierarchical framework. This integration of qualitative discovery and quantitative classification provides a robust methodology that directly links the identification of needs to their strategic prioritization. By employing this framework, we shift the research paradigm from a technology-acceptance focus to a satisfaction-driven, user-centered design analysis [34], setting the stage for an evidence-based roadmap for next-generation AIGC service development.

## Methodology

### Scope of AIGC services

To ensure clarity and precision in our investigation, it is essential to define the boundaries of “AIGC services” within the context of academic research. In this study, AIGC services are defined not merely as Large Language Models (LLMs) like ChatGPT, but as a broader ecosystem of generative AI tools specifically applied to research workflows.

Specifically, the scope of AIGC services in this study encompasses two primary categories:

General-Purpose LLMs: Tools such as ChatGPT (OpenAI), Claude (Anthropic), and Gemini (Google), which researchers use for broad tasks like drafting, brainstorming, and coding assistance.

Specialized Academic AI Tools: Vertical applications built upon generative AI technologies, such as Elicit, Scite, and ResearchRabbit. These tools leverage generative models for specific academic tasks, including semantic literature review, citation analysis, and data interpretation.

Functional Inclusions and Exclusions:Included Functions: The study covers generative capabilities such as automated text generation (writing/polishing), code generation, semantic summarization of literature, and AI-driven image/data visualization.

Excluded Tools: Traditional digital tools that do not utilize generative AI algorithms are excluded from this study. For instance, standard reference management software (e.g., legacy versions of EndNote) or keyword-based search engines (e.g., Web of Science) are not considered AIGC services unless they explicitly integrate generative AI features.

By defining this scope, we ensure that the participants’ responses regarding “needs” and “satisfaction” are grounded in the capabilities of current generative technologies rather than traditional software.

### Research design

This study employed an exploratory sequential mixed-methods design [35] to systematically investigate researchers’ hierarchical needs for AIGC services. The choice of an exploratory sequential mixed-methods design is grounded in the emergent and rapidly evolving nature of AIGC technology in academia. Since researchers’ needs for AIGC are often latent or unarticulated—beyond simple text generation—a purely quantitative approach might overlook novel or context-specific demands. Therefore, the initial qualitative phase (Phase 1) is essential to discover and define the full spectrum of potential service attributes from the bottom up. The subsequent quantitative phase (Phase 2) is then critical to empirically validate these attributes and classify them hierarchically across a representative sample. This design ensures that the final prioritization framework is both deeply contextualized in actual research practices and statistically generalizable, directly addressing RQ1 and RQ2.The research proceeded in two distinct phases:

Phase 1 (Qualitative Exploration):We first conducted semi-structured interviews with researchers to explore and identify a comprehensive list of desired AIGC service functions. The interview data were then analyzed using computational thematic analysis with BERTopic to distill core demand items with enhanced objectivity and reproducibility.

Phase 2 (Quantitative Classification):The demand items identified in Phase 1 were used to construct a Kano model-based questionnaire. A large-scale survey was then conducted to quantitatively classify these demands into a needs hierarchy and assess their relative importance in influencing user satisfaction.

### Participants and procedure

This study utilized a two-phase data collection process involving semi-structured interviews and a quantitative Kano questionnaire. A stratified sampling approach was broadly employed across both phases to ensure the collected data represented a comprehensive cross-section of the academic community, accounting for variations in research practices across disciplines and career stages.

#### Phase 1: Qualitative data collection for demand identification.

For the qualitative phase, we employed a purposive sampling strategy to recruit 45 researchers, aiming to maximize variation across discipline, career stage, and AIGC experience. The expert selection criteria were as follows:(1) Active researcher (Ph.D. candidate or above) with a minimum of two peer-reviewed publications. (2) Verifiable experience using at least one major AIGC tool (e.g., ChatGPT, Elicit, Scite) for research tasks within the last six months. (3) Representation across both STEM and HSS disciplines to capture diverse methodological needs. (4) A balance of career stages (early, mid, senior) to reflect differing workflow pressures. We initiated recruitment through university mailing lists and academic social networks (e.g., ResearchGate). Potential participants who responded were then screened against these criteria via a short pre-interview questionnaire to ensure their suitability for the study.

Semi-structured interviews were conducted online between 10 May 2025 and 30 May 2025. Each interview lasted approximately 45–60 minutes and was audio-recorded and transcribed verbatim for analysis. The interview protocol was structured around the five key stages of the research lifecycle: research ideation, research design, data analysis, manuscript writing, and post-publication activities. Participant demographics are detailed in Table 1. As detailed in Table 1, our purposive sampling strategy ensured a balanced and diverse sample across key dimensions such as discipline, career stage, and AIGC experience, which was crucial for achieving theoretical saturation.

**Table 1:** Demographic profile of interview participants (N = 45).

Characteristic	Category	N	Percentage	Brief Description
Primary Discipline Area	STEM(Science,Technology, Engineering, & Math)	22	48.9%	To capture perspectives from empirical and experimental sciences.
	-Physical & Engineering Sciences	12	26.7%	e.g., Physics,Computer Science
	-Life & Medical Sciences	10	22.2%	e.g., Biology,Clinical Research
	HSS (Humanities & Social Sciences)	23	51.1%	Tocapture perspectives from interpretive and text-intensive fields.
	-Social Sciences	13	28.9%	e.g., Sociology,Economics, Education
Academic Career Stage	Early-Career (Ph.D. Candidate)	15	33.3%	Represents digitally-native researchers facing high publication pressure.
	Mid-Career (Postdoctoral Fellow/ Asst. Professor)	16	35.6%	Represents researchers establishing their academic independence.
	Senior-Career (Associate/ Full Professor)	14	31.1%	Represents established researchers with supervisory roles.
AIGC Experience Level	Novice (Limited or no prior use, basic awareness)	10	22.2%	To understand barriers to adoption and initial needs.
	Intermediate (Regular use for specific tasks)	25	55.6%	Represents the mainstream user base and their routine demands.
	Expert (Advanced use, integrated into workflow)	10	22.2%	To explore cutting-edge use cases and future service potential.
Geographic Distribution	Universities in Eastern China	24	53.3%	Represents major research hubs with high resource access.
	Universities in Central/Western China	21	46.7%	To ensure inclusion of perspectives from diverse institutional contexts.

#### Phase 2: Quantitative data collection for demand prioritization.

For the quantitative phase, the goal was to recruit a large and representative sample to ensure the generalizability of the Kano analysis. The online questionnaire was distributed from 8 June 2025 to 26 June 2025 through targeted academic forums and mailing lists, again employing a stratified approach to reach researchers across various disciplines and career stages. A total of 500 questionnaires were distributed. After data cleaning, responses that were incomplete or invalid (e.g., those with a completion time of under five minutes, indicating a lack of serious engagement) were removed. This process yielded 412 valid responses, resulting in a high effective response rate of 82.4%.

The demographic characteristics of the survey respondents are summarized in Table 2. A comparison with national researcher population statistics confirms that our sample is highly representative in terms of disciplinary field and academic position, with deviations being minimal. This strengthens the external validity of our quantitative findings.

**Table 2:** Comparison of survey sample demographics (N = 412) with national researcher population.

Characteristic	Category	Our Sample (N = 412)	Population Benchmark^1^	Deviation
Disciplinary Field	STEM	53.4%(n = 220)	54.2%	−0.8%
	- Natural Sciences	21.8% (n = 90)	22.1%	−0.3%
	- Engineering	24.3% (n = 100)	25.3%	−1.0%
	- Agri. & Med. Sci.	7.3% (n = 30)	6.8%	+0.5%
	HSS	37.4% (n = 154)	36.5%	+0.9%
	- Social Sciences	21.8% (n = 90)	21.4%	+0.4%
	- Humanities	15.5% (n = 64)	15.1%	+0.4%
	Other(e.g.,Art, Interdisciplinary)	9.2% (n = 38)	9.3%	−0.1%
Academic Position	Ph.D. Candidate	30.8% (n = 127)	30.5%	+0.3%
	Postdoc/Lecturer	34.7% (n = 143)	35.0%	−0.3%
	Associate Professor	22.1% (n = 91)	22.5%	−0.4%
	Full Professor	12.4% (n = 51)	12.0%	+0.4%

Note: Population benchmark data were adapted from the China Statistical Yearbook on Science and Technology 2024, published by the National Bureau of Statistics of China.

The Kano questionnaire was constructed based on the 15 core service demands identified in Phase 1. The survey was administered online, and participants were presented with an information sheet at the beginning outlining the study’s purpose and their rights. Completion of the survey was considered implied consent.

### Data analysis

The data analysis process was also conducted in two sequential phases, directly corresponding to the qualitative and quantitative data collected.

#### Phase 1: Identifying core demands via computational thematic analysis.

To systematically identify core service demands from the 45 interview transcripts, we used BERTopic [13], a state-of-the-art topic modeling technique. This method was selected for its ability to understand the contextual meaning of sentences, a significant advantage over traditional word-frequency-based models like LDA, thus allowing for a more accurate interpretation of researcher needs [36].

Our analytical pipeline integrated computational rigor with qualitative validation and proceeded as follows:

1. Corpus Preparation and Embedding: Interview transcripts were preprocessed and segmented into sentences. Each sentence was then converted into a rich numerical vector using a pre-trained Sentence-BERT model to capture its semantic meaning.
2. Topic Clustering: We applied UMAP for dimensionality reduction and HDBSCAN for clustering to group semantically similar sentences into distinct topics, each representing a potential user demand.
3. Human Validation and Thematic Synthesis: The computationally generated topics were then subjected to rigorous human validation. Two researchers independently analyzed the sentence clusters in NVivo 12 to confirm their conceptual coherence and assign a definitive thematic label to each. Disagreements were resolved through discussion to reach consensus, resulting in high inter-rater reliability (Cohen’s Kappa = 0.89).

This mixed-method approach ensured that the final 15 service demands were both data-driven and contextually validated.

#### Phase 2: Prioritizing service demands using the Kano model.

Following the identification of 15 core service demands in Phase 1, the research progressed to a quantitative stage aimed at classifying and prioritizing these demands from the user’s perspective. The central challenge at this juncture was to understand not just what researchers need, but how the fulfillment or absence of these needs would impact their overall satisfaction. Simple ranking or rating scales (e.g., Likert scales) are insufficient for this purpose as they fail to capture the asymmetric and non-linear relationship between feature performance and user satisfaction [10].

Therefore, we adopted the Kano model as our analytical framework. This model provides a robust and theoretically grounded methodology for categorizing user preferences into distinct quality attributes: Must-be (M), One-dimensional (O), Attractive (A), Indifferent (I), and Reverse (R). Its suitability for this study is threefold: First, it is specifically designed for product and service development, making it highly relevant for assessing potential AIGC features [37]. Second, it moves beyond simple importance ratings to reveal the latent satisfaction structure, which is crucial for making strategic design decisions. For instance, it helps distinguish foundational “must-have” features from “attractive attribute” features that can create a competitive advantage. Third, its unique functional/dysfunctional questioning format provides a solid foundation for translating the abstract demands identified via BERTopic into concrete, testable propositions.

The implementation of the Kano model followed a structured protocol:

Questionnaire Design: For each of the 15 demands, a pair of questions was formulated. The functional question measured the user’s response if the service demand was met (e.g., “If the AIGC tool could automatically format your references, how would you feel?”). The dysfunctional question measured the response if the demand was not met (e.g., “If the AIGC tool could NOT automatically format your references, how would you feel?”). Respondents answered on a 5-point scale from “1. I like it that way” to “5. I dislike it that way.”

Survey Administration and Sampling: The questionnaire was distributed to a broader population of researchers, achieving a total of 412 valid responses. A stratified sampling strategy was employed to ensure representation across different academic disciplines and career stages, thereby enhancing the generalizability of our findings.

Data Analysis and Classification: Responses were analyzed using the standard Kano evaluation table to classify each demand for each respondent. To derive an aggregate classification and assess relative importance, we calculated the Better-Worse coefficients [38]. This method provides a more nuanced analysis than simple frequency counts of categories. The coefficients are calculated as:

Equation 1: Better-Worse Coefficient Calculation:


$$Better(CS+)=\frac{A+O}{A+O+M+I}$$ (1)

$$Worse(CS-)=-\frac{O+M}{A+O+M+I}$$ (2)

The Better coefficient (or Customer Satisfaction coefficient, CS+) measures the degree to which satisfaction increases if a demand is met, with higher values indicating a stronger positive impact. The Worse coefficient (or Customer Dissatisfaction coefficient, CS-) measures the degree to which satisfaction decreases if a demand is not met, with more negative values indicating a stronger negative impact. Plotting these coefficients on a 2D-grid allowed for a robust, empirically validated classification of the 15 demands, providing a solid foundation for our hierarchical needs framework.

### Ethical considerations

This study, which prospectively recruited human participants, received ethical approval from the Biomedical Ethics Committee at Qufu Normal University (Approval No: 2025110). All procedures were conducted in accordance with the relevant ethical guidelines.

The recruitment for Phase 1 (qualitative interviews) occurred between 10 May 2025 and 30 May 2025. Prior to each interview, participants received a detailed explanation of the study’s objectives and procedures, after which they provided written informed consent.

The recruitment for Phase 2 (quantitative survey) was conducted from 8 June 2025 to 26 June 2025. For this phase, the ethics committee waived the need for written consent due to the anonymous and low-risk nature of the online survey. Participants were presented with an information sheet at the beginning of the questionnaire detailing the study’s purpose, the voluntary nature of their participation, and their right to withdraw at any time. Proceeding with the survey was considered implied consent.

## Results

This chapter presents the findings from our mixed-methods analysis. We first report the core AIGC service demands identified through qualitative analysis. Subsequently, we present the quantitative classification of these demands based on the Kano model survey, including an analysis of statistically significant differences across key researcher demographics.

### Identifying core service demands across the research lifecycle

To distill a comprehensive list of service demands from the 45 interview transcripts, we conducted a computational thematic analysis using BERTopic. This process pinpointed 15 distinct service demands. These distilled demands, which formed the foundational items for our subsequent quantitative Kano survey, are summarized in Table 3. For each theme, the table lists the most representative words generated by the model to aid interpretation; these words are derived from a class-based TF-IDF (c-TF-IDF) algorithm, which serves as a descriptive labeling function within the BERTopic framework, not as the basis for clustering.

**Table 3:** Core AIGC service demands identified via computational thematic analysis.

Identified Demand Theme	Representative Topic Words	Representative User Quote
Literature Gap Identification	gap(0.92),literature (0.87)	“An AI tool that spots contradictions between studies would be invaluable. Identifying gaps is the most critical step.”
Statistical & Ethical Rigor	sample size(0.93),ethics(0.90)	“Calculating the required sample size is often tricky; an AI assistant would be a huge help to ensure our study is sufficiently powered.”
Visualization & Interpretation	visualize(0.94),analysis(0.92)	“The ability to automatically visualize data and create publication-quality charts from results is highly desirable.”
Accuracy & Integrity	citation(0.95),plagiarism(0.93)	“Ensuring citation accuracy is a must-be feature; any errors here are unacceptable for maintaining academic integrity.”
Dissemination & Impact	journal(0.96),reviewers(0.92)	“Suggesting a suitable journal is incredibly helpful, but drafting a structured response to reviewer comments is one of the most stressful tasks.”

The analysis reveals a clear progression of needs: from creative and exploratory support during ideation, to methodological and ethical rigor in research design, efficiency and interpretive power in data analysis, precision and integrity in manuscript writing, and finally, impact amplification during post-publication activities. These distilled demands formed the foundational items for our subsequent quantitative Kano survey.

The analysis of demands regarding research design reveals that researchers seek support in translating abstract ideas into rigorous, actionable plans. Specific demands include: first, the service should recommend appropriate research methodologies (e.g., quantitative, qualitative, experimental) based on the research question and field-specific conventions; second, it needs to assist in constructing detailed experimental or study protocols, including suggestions for sample size calculation, control groups, and variable selection; third, it should be capable of identifying and suggesting validated measurement instruments, surveys, or data collection techniques to ensure the reliability and validity of the research.

The analysis of demands concerning data analysis shows that researchers desire AIGC services to enhance both the efficiency of processing and the depth of interpretation. Specific demands include: first, the service must be able to generate executable code for data cleaning, statistical analysis, and modeling in common programming languages like Python or R, based on natural language descriptions of the desired analysis; second, it should assist in the interpretation of complex statistical outputs, explaining the meaning of results in clear, accessible terms; finally, the service should be able to automatically create accurate and publication-ready data visualizations, such as graphs and charts, directly from raw data tables.

The analysis of demands for manuscript writing and revision indicates that researchers’ overall demands are for an intelligent writing partner that can streamline the path from draft to publication. Specific demands include: first, the service should assist in drafting sections of the manuscript, such as the literature review or methodology, while maintaining a consistent academic tone and adhering to user-provided outlines; second, it must offer advanced proofreading and copy-editing capabilities, checking for grammatical accuracy, clarity, and style consistency; third, it should automate the tedious process of formatting citations and bibliographies according to specific journal guidelines (e.g., APA, MLA, Chicago); finally, the service should be able to analyze feedback from peer reviewers and suggest specific revisions or generate draft responses.

The analysis of demands regarding post-publication activities shows that researchers wish for services that can help maximize the impact and reach of their work. Specific demands include: first, the service should have the capability to generate accessible summaries of the research for diverse audiences, such as lay summaries, press releases, or social media content; second, it must be able to track the dissemination and impact of the publication, including monitoring citations, mentions in news media, and discussions on academic platforms; finally, the service should identify potential future collaborators or relevant funding opportunities by analyzing the topic of the published work and its reception within the scholarly community.

### Hierarchical classification of AIGC service demands

**Fig 2:**
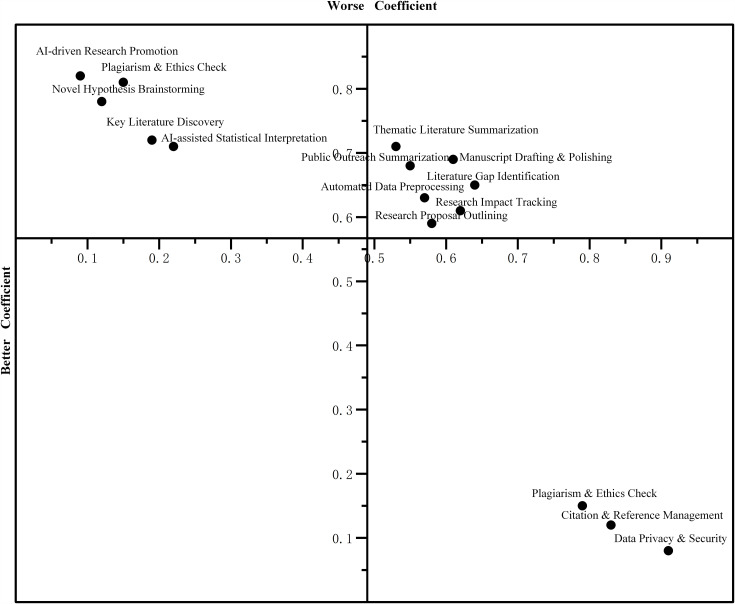
Kano Model classification grid based on better-worse coefficients.

Based on the attributes identified in the qualitative phase, a Kano survey was administered to 412 researchers. allowed us to classify each service attribute into a distinct hierarchical category. The Better-Worse coefficients, which determine the classification, are detailed in Table 4, and the final categorization is visualized in Fig 2. The results reveal a clear structure comprising three Must-be, seven One-dimensional, five Attractive attributes. Notably, three functions (e.g., user interface customization, gamification features) were classified as Indifferent attributes, indicating they do not significantly impact user satisfaction; for brevity, these are not detailed further in the analysis.

**Table 4:** Better-worse coefficients and demand classification for AIGC services.

Demand item	Better Coefficient	Worse Coefficient	Definition
Novel Hypothesis Brainstorming	0.78	−0.12	Utilizing AI to generate interdisciplinary ideas or innovative concepts by connecting disparate research topics.
Literature Gap Identification	0.65	−0.64	Systematically analyzing existing studies to pinpoint unexplored areas or contradictions that warrant further investigation.
Research Proposal Outlining	0.59	−0.58	Structuring the logic and flow of a research grant or thesis proposal, including objectives and methodology.
Thematic Literature Summarization	0.71	−0.53	Aggregating and synthesizing key findings from multiple papers into a coherent thematic review.
Citation & Reference Management	0.12	−0.83	Formatting citations according to specific journal styles and checking for metadata accuracy.
Key Literature Discovery	0.72	−0.19	Intelligent retrieval of the most influential or relevant papers based on semantic meaning rather than just keywords.
Automated Data Preprocessing	0.63	−0.57	Cleaning, formatting, and structuring raw data (e.g., text or spreadsheets) to make it ready for analysis.
AI-assisted Statistical Interpretation	0.71	−0.22	Explaining complex statistical outputs (e.g., regression coefficients, p-values) in plain academic language.
Data Privacy & Security	0.08	−0.91	Features ensuring that user-uploaded data is encrypted and not used for model training without consent.
Manuscript Drafting & Polishing	0.69	−0.61	Generating initial drafts or refining the language of existing text to improve fluency and academic tone.
Plagiarism & Ethics Check	0.15	−0.79	Scanning text for potential plagiarism risks and flagging content that may violate ethical guidelines.
Smart Journal Recommendation	0.81	−0.15	Analyzing the manuscript’s topic, scope, and quality to suggest suitable target journals.
AI-driven Research Promotion	0.82	−0.09	Creating content (e.g., Twitter threads, blog posts) to disseminate published research to a wider audience.
Research Impact Tracking	0.61	−0.62	Monitoring and predicting the academic or social impact of research outputs (citations, altmetrics).
Public Outreach Summarization	0.68	−0.55	Simplifying complex academic jargon into “plain language summaries” for the general public or policymakers.

### Must-be attributes: The foundation of trust and usability

In our analysis, three core functions were identified as Must-be attributes, characterized by their extremely high negative impact when absent. As shown in Table 4, Data Privacy & Security (Worse: −0.91), Citation & Reference Management (Worse: −0.83), and Plagiarism & Ethics Check (Worse: −0.79) all have dissatisfaction coefficients approaching −1.0, confirming their status as foundational expectations.

This pattern indicates they are fundamental user expectations [39]. Their seamless performance is considered a basic requirement and thus does not generate significant satisfaction. However, any failure or absence of these functions would cause extreme user dissatisfaction, severely damage the tool’s credibility, and render it unusable for serious academic work [40]. They are the non-negotiable bedrock of academic integrity and workflow security [41], forming the user’s foundational layer of trust.

### One-dimensional attributes: The core engine of productivity

Seven functions were classified as One-dimensional attributes, representing the tool’s core productivity engine. These include features like Automated Data Preprocessing (0.63, −0.57), Manuscript Drafting & Polishing (0.69, −0.61), and Thematic Literature Summarization (0.71, −0.53). Other key functions in this category are Research Impact Tracking (0.61, −0.62), Literature Gap Identification (0.65, −0.64), Research Proposal Outlining (0.59, −0.58), and Public Outreach Summarization (0.68, −0.55).

These attributes exhibit a clear, linear relationship with user satisfaction: the better they perform (e.g., higher accuracy, faster speed, more comprehensive results), the higher the user’s satisfaction. Conversely, poor performance directly leads to user frustration. These functions directly address the most time-consuming and labor-intensive aspects of the research lifecycle, and continuous improvement in their quality and efficiency is the most direct path to increasing the tool’s perceived value and utility.

### Attractive attributes: The drivers of delight and innovation

Five functions emerged as Attractive attributes, acting as powerful differentiators. These are AI-driven Research Promotion (0.82, −0.09), Smart Journal Recommendation (0.81, −0.15), Novel Hypothesis Brainstorming (0.78, −0.12), Key Literature Discovery (0.72, −0.19), and AI-assisted Statistical Interpretation (0.71, −0.22).

These features are defined by their high satisfaction coefficients (high Better scores) and very low dissatisfaction coefficients (Worse scores close to 0). Their absence would likely go unnoticed, as users do not currently expect them from a standard research tool. However, their presence creates a “wow” effect, delighting users and significantly enhancing the user experience. These attributes transform the tool from a simple assistant into an intelligent intellectual partner, fostering creativity and providing unexpected value. Investing in these innovative features can create a strong competitive advantage and foster deep user loyalty.

### Cross-group differences in AIGC needs: A statistical analysis

To move beyond a monolithic view of “the researcher,” we investigated how needs differ across disciplines (STEM vs. HSS) and career stages (Early-Career vs. Established). We employed independent samples t-tests to compare the importance scores derived from the Better-Worse coefficients for key One-dimensional and Attractive attributes. The results are summarized in Table 5.

**Table 5:** Statistical comparison of AIGC needs across disciplines and career stages.

Attribute Name	STEM Mean (SD)	HSS Mean (SD)	p-value(Cohen’s d)	Early-Career Mean (SD)	Established Mean (SD)	p-value (Cohen’s d)
Automated Data Preprocessing	0.75(0.21)	0.48(0.30)	<.001(d = 1.01)*	0.65(0.25)	0.61(0.28)	.189(n.s.)
Thematic Literature Summarization	0.62(0.25)	0.82(0.18)	<.001(d = −0.91)*	0.73(0.21)	0.69(0.24)	.145(n.s.)
Novel Hypothesis Brainstorming	0.71(0.28)	0.77(0.24)	.061(n.s.)	0.70(0.29)	0.85(0.21)	<.01(d = −0.59)*
Smart Journal Recommendation	0.75(0.22)	0.80(0.20)	.075(n.s.)	0.88(0.15)	0.74(0.25)	<.001(d = 0.68)*

* Note: Importance scores were measured on a scale from 0 to 1. n.s. = not significant. Cohen’s d values are interpreted as small (0.2), medium (0.5), and large (0.8). Indicates statistical significance at p < .05.

Detailed comparisons of attribute importance across disciplines and career stages are presented in Table 5. Our analysis revealed significant cross-disciplinary differences. For instance, STEM researchers placed a substantially higher value on ‘Automated Data Preprocessing’ compared to their HSS counterparts (M_STEM = 0.75 vs. M_HSS = 0.48, p < .001). The large effect size (Cohen’s d = 1.01) underscores the practical importance of this difference. Conversely, HSS researchers showed a significantly stronger demand for ‘Thematic Literature Summarization’ (M_HSS = 0.82 vs. M_STEM = 0.62, p < .001), also with a large effect size (Cohen’s d = −0.91).

Regarding career stage, early-career researchers demonstrated a significantly higher need for ‘Smart Journal Recommendation’ (M_ECR = 0.88 vs. M_Est = 0.74, p < .001), with a medium-to-large effect size (Cohen’s d = 0.68), likely reflecting greater pressure for publication in high-impact venues.

## Discussion

The discussion section is structured to synthesize and interpret the findings presented in the results.It begins by identifying the overarching characteristics of researchers’ demands for AIGC services. Subsequently, it uses these characteristics as an analytical framework to interpret the Kano model’s need hierarchy. Finally, it elaborates on the theoretical and practical implications of the study.

### The nature of AIGC demands: A synthesis of findings

Synthesizing our qualitative and quantitative findings, we can distill the overarching characteristics of researchers’ demands for AIGC services into four analytical dimensions. These dimensions, which help explain the underlying logic of the Kano classifications, can be organized into two pairs: immediacy and adaptability (concerning the nature of interaction) and lifecycle integration and progressive sophistication (concerning the nature of the workflow).

Horizontally, immediacy refers to the need for real-time, on-demand support for time-sensitive tasks, such as instant literature summarization or brainstorming. Adaptability highlights the necessity for AIGC tools to be flexible and context-aware, tailoring their outputs to specific research fields, individual user expertise, and evolving project requirements.

Vertically, lifecycle integration signifies the need for seamless, interconnected tools that support the entire research workflow—from initial ideation to final publication and outreach. This holistic approach prevents fragmentation and enhances efficiency. Progressive sophistication captures the expectation that AIGC services should evolve from handling basic, administrative tasks (e.g., citation formatting) to assisting with complex, higher-order cognitive functions (e.g., generating novel hypotheses), thereby becoming true intellectual partners.These four characteristics collectively frame researchers’ expectations and provide a robust foundation for interpreting their prioritization of AIGC service demands.

These four dimensions, derived from our results, collectively frame researchers’ expectations and provide a robust foundation for interpreting their prioritization of AIGC service attributes in the following section.

### Cross-demand differential analysis: Uncovering nuancesin the scholarly community

A key contribution of this study lies in its ability to move beyond a monolithic view of “the researcher.” By analyzing our stratified sample, we uncover subtle but significant differences in needs across disciplines and career stages, adding a crucial layer of practical value to the Kano model.

Disciplinary Divides: While Must-be attributes like data security were universally prioritized, we observed a notable divergence in One-dimensional needs. Our qualitative observations were strongly supported by independent samples t-tests.For instance, researchers in STEM fields placed a significantly higher value on Automated Data Preprocessing and AI-assisted Statistical Interpretation compared to their counterparts in the humanities and social sciences (HSS)(p < .001, as shown in Table 5). Conversely, HSS scholars expressed a significantly stronger need for Thematic Literature Summarization (p < .001), reflecting their discipline’s emphasis on interpretive and narrative-driven scholarship. This finding provides empirical evidence for the “two cultures” in the age of AI, demonstrating that technological needs are deeply shaped by the underlying epistemological paradigms of different disciplines.

Career Stage Considerations: The perceived importance of certain Attractive attributes varied significantly with career progression. Early-career researchers (ECRs) rated Smart Journal Recommendation and AI-driven Research Promotion as highly attractive. This likely reflects the immense pressure on ECRs to publish in high-impact venues and build their academic profile. Our statistical analysis confirms this, showing that ECRs placed significantly higher importance on Smart Journal Recommendation than established researchers (p < .001). In contrast, established researchers showed greater enthusiasm for Novel Hypothesis Brainstorming, an Attractive attribute that aligns with their focus on pioneering new research directions and securing large-scale funding.Likewise, this preference was statistically significant (p < .01), indicating that senior scholars value AIGC’s potential as an “intellectual partner” for stimulating innovation. These findings suggest that AIGC services should not be “one-size-fits-all” but should offer customizable feature sets tailored to the specific workflows and career pressures of different user segments.

### Interpreting the needs hierarchy: The logic behind prioritization

This section synthesizes the Kano classification to address **RQ2** and **RQ3**, interpreting the hierarchy through the theoretical lenses of Human-Centered AI (HCAI) and Task-Technology Fit (TTF). The prioritization reflects a logical progression from trust to efficiency, and finally to co-creation.

Must-be Attributes: From “Basic Needs” to “Foundation of Trust”

Addressing RQ2 regarding the foundational requirements, the classification of Data Privacy, Citation Accuracy, and Plagiarism Checks as Must-be attributes reveals a critical theoretical insight: for academic users, trust is the prerequisite for adoption. From the perspective of Human-Centered AI (HCAI), these attributes represent the “Reliability and Safety” layer. Unlike casual users who might tolerate hallucinations in a chatbot, researchers operate in a high-stakes environment where validity is paramount. The extreme negative impact of these attributes’ absence (high Worse coefficients) indicates that without this foundation of trust, the “human-in-the-loop” collaboration envisioned by HCAI collapses. AIGC services must first validate their role as reliable stewards of academic integrity before they can be accepted as productivity tools.

One-dimensional Attributes: From “Useful Features” to “Engine of Efficiency”

Regarding the performance drivers identified in RQ3, the One-dimensional attributes—such as Automated Data Preprocessing and Literature Summarization—act as the “Engine of Efficiency.” Their strong linear correlation with user satisfaction directly aligns with the Task-Technology Fit (TTF) theory. These attributes target the most labor-intensive, cognitive bottlenecks in the research lifecycle. The positive Better coefficients confirm that the value of AIGC here lies in “cognitive offloading”—freeing researchers from repetitive tasks to focus on higher-order thinking. This suggests that for these functions, the HCAI goal is “Augmentation”: simply doing what the human does, but faster and more accurately, thereby directly enhancing research throughput.

Attractive Attributes: From “Wow Factors” to “Catalyst for Innovation”

Finally, the Attractive attributes provide a forward-looking answer to RQ3, highlighting the evolving role of AIGC from a mere tool to an intellectual partner. Features like Novel Hypothesis Brainstorming and Smart Journal Recommendation go beyond efficiency; they act as a “Catalyst for Innovation.” In the HCAI framework, this represents the highest level of interaction: “Co-creation.” These features offer capabilities that extend human cognitive limits, exploring solution spaces that a researcher might not reach alone. The fact that these are Attractive (unexpected delights) rather than Must-be suggests that we are currently at a transitional moment where AIGC is beginning to reshape the epistemological process of discovery itself, offering a glimpse into a future where AI actively participates in knowledge generation rather than just information processing.

### Theoretical and practical implications

The synthesis of our findings offers significant theoretical and practical implications for the development and adoption of AIGC in academia.

From a theoretical perspective, this research, grounded in the Kano model and principles of Human-Computer Interaction (HCI) [8], analyzes the requirements for constructing AIGC services for researchers. Theoretically, this study not only integrates research on AI in science [42], scholarly communication, and technology acceptance models [24], but also promotes further development in these fields. Firstly, it highlights that AIGC model construction must move beyond generic performance and focus on domain-specific fine-tuning and the traceability of generated claims to verifiable sources [43]. Secondly, it enriches the design paradigm for scholarly tools by advocating for a holistic, research-lifecycle approach [23], where AIGC services are integrated from initial ideation through to final publication, rather than serving as point solutions for isolated tasks. Finally, this study refines the conceptual framework of trust in AI for expert domains by emphasizing that for researchers [44], trust is not merely about system reliability but is intrinsically linked to verifiability, citation accuracy, and the transparency of the AI’s reasoning process [45].

From a practical perspective, our findings suggest that the construction of AIGC services for researchers should follow a phased approach, logically derived from our hierarchical needs framework.

**Fig 3:**
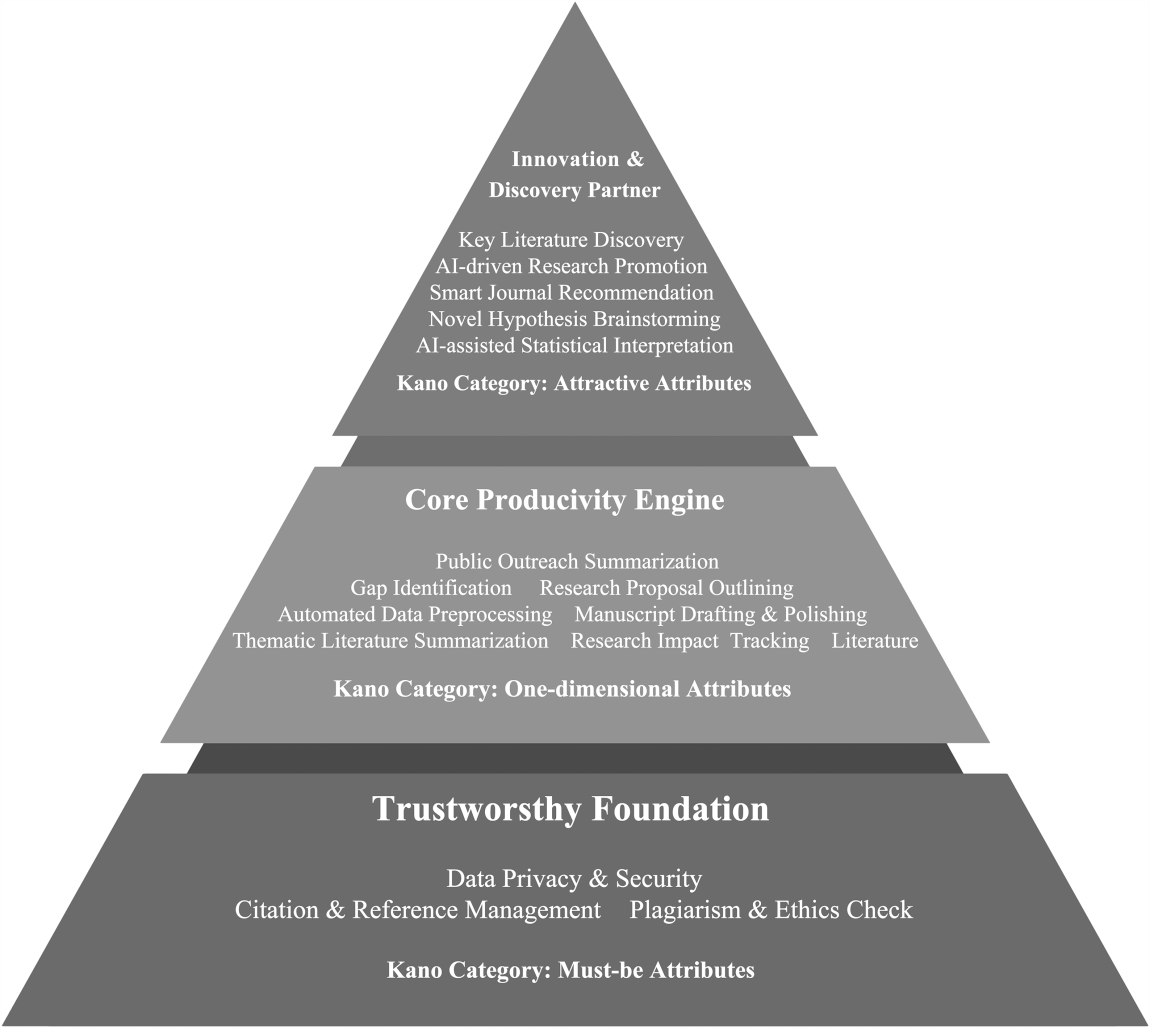
A phased roadmap for AIGC service development based on the hierarchical needs model.

Our findings offer a clear, actionable blueprint for developers and providers of AIGC services, guiding them on what to build and in what order. Based on the hierarchical nature of researcher demands revealed by the Kano model, we propose a three-phase development roadmap, visually summarized in Fig 3. This roadmap prioritizes features to maximize user adoption and satisfaction.

Phase 1: Build the Trustworthy Foundation. The development priority should unequivocally be on the Must-be attributes. As shown in the foundational layer of our model (Fig 3), features like “Data Privacy & Security,” “Citation Accuracy,” and “Fact-Checking Support” are non-negotiable. They form the bedrock of user trust. An AIGC platform that fails in this domain will be rejected by the academic community, regardless of how advanced its other features are. These are the “table stakes” for entering the scholarly market.

Phase 2: Develop the Core Productivity Engine. Once the foundation of trust is established, development efforts should focus on One-dimensional attributes. These features, such as “Automated Literature Review” and “Manuscript Drafting,” are the workhorses of the AIGC service. They directly address researchers’ core pain points related to efficiency. The quality and performance of these tools will be the primary driver of user satisfaction and a key differentiator in a competitive market. Investment in this phase yields a direct, proportional return in user value.

Phase 3: Emerge as an Innovation & Discovery Partner. Finally, with a robust and trustworthy platform in place, providers can introduce Attractive attributes to truly delight users and foster loyalty. Features like “Hypothesis Generation” and “Smart Journal Recommendation” position the AIGC tool not just as an assistant, but as a creative partner in the research process. While users may not explicitly ask for these “wow” features, their inclusion can create significant competitive advantage and transform the user experience from merely functional to genuinely inspiring. This phased approach ensures that development resources are allocated strategically, first securing user trust, then delivering core value, and finally, aiming for innovation.

For Academic Institutions: Strategic Procurement and Training.

For universities and research libraries, this hierarchical framework serves as a guide for resource allocation, policy making, and user training.

Procurement and Evaluation Standards: When evaluating AIGC tools for institutional subscription, decision-makers should not be dazzled by “Attractive” features (e.g., creative brainstorming) if the tool fails on “Must-be” criteria. Our study suggests that Data Privacy and Citation Integrity must be the veto criteria in any procurement tender. Institutions should demand rigorous “Trust Audits” for these specific attributes before deployment.Differentiated Training Programs: Recognizing the demographic differences in needs, libraries should move away from generic “Introduction to AI” workshops.

For Early-Career Researchers: Training should focus on leveraging Attractive attributes (e.g., Smart Journal Recommendation) to navigate publication pressures, while simultaneously reinforcing ethical boundaries (Must-be attributes) to prevent academic misconduct.

For HSS Researchers: Workshops should emphasize One-dimensional attributes like Thematic Literature Summarization to assist with narrative-heavy workflows.

For STEM Researchers: Support should focus on Data Preprocessing and Statistical Interpretation tools to enhance experimental rigor.

### Limitations and future directions

While this study offers a pioneering framework for AIGC demands, several limitations warrant consideration for proper interpretation.

Geographical and Cultural Specificity: First, as noted, the sample is drawn exclusively from Chinese institutions. The high demand for “Smart Journal Recommendation” may be partially driven by the specific quantitative assessment metrics prevalent in the Chinese academic evaluation system. The prioritization of needs may shift in academic cultures with different incentive structures (e.g., tenure systems in North America or Europe).

The “Snapshot” Nature of Kano Categories: Second, the Kano model captures user satisfaction at a specific point in time. According to the lifecycle theory of attractive quality, features that are currently “Attractive” (e.g., AI brainstorming) tend to degrade into “Must-be” attributes over time as users become habituated to them. This study represents a static snapshot of a highly dynamic technological landscape; what delights researchers today may be expected as a standard tomorrow.

Self-Reporting Bias: Finally, our methodology relies on stated preferences rather than behavioral data. There may be a “say-do gap” where researchers express a desire for high-level cognitive support (e.g., Hypothesis Brainstorming) to appear innovative, but in daily practice, primarily utilize the tool for basic efficiency tasks (e.g., Polish). Future studies would benefit from corroborating survey data with actual usage logs.

Based on our findings and limitations, we propose three critical directions for future inquiry to advance the understanding of AIGC in academia.

Longitudinal Tracking of Demand Evolution: Future research should adopt a longitudinal design to track how AIGC attributes migrate across Kano categories. Determining the “decay rate” of Attractive attributes would help developers predict when a novel feature will become an industry standard, allowing for more proactive innovation cycles.

Cognitive Impact and “Human-in-the-Loop” Dynamics: While our study identifies what researchers want, it does not address the consequences of fulfilling these wants. Specifically, does the heavy reliance on “One-dimensional” attributes (e.g., Automated Literature Summarization) lead to a “cognitive offloading” that might atrophy researchers’ critical synthesis skills? Future work should investigate the long-term epistemological impact of using these tools: does AI-assisted efficiency come at the cost of intellectual depth?

From “User Needs” to “Human-AI Teaming”: As AIGC capabilities evolve from text generation to multi-modal reasoning, the research focus must shift from “acceptance” to “collaboration.” Future studies should explore how AIGC can be designed to function not just as a subordinate assistant, but as a dyadic team member. How do researchers negotiate agency and control when an AI suggests a novel hypothesis? Research into these human-AI teaming dynamics will be essential for designing the next generation of scholarly tools that augment, rather than replace, human scientific inquiry.

## Conclusion

This study successfully deconstructed the multifaceted demands of researchers for AIGC services, revealing a clear three-tiered hierarchy of needs: foundational Must-be attributes centered on trust, core One-dimensional attributes driving productivity, and innovative Attractive attributes fostering discovery. Our findings provide an evidence-based roadmap for moving beyond generic ‘writing machines’ toward human-centered, lifecycle-integrated AIGC partners that can genuinely enhance scholarly research.

While this research lays a crucial foundation, its focus on the academic research community necessarily sets the stage for broader future inquiry.Subsequent research should, for instance, adopt a multi-stakeholder approach, incorporating the perspectives of key players such as academic librarians, research integrity officers, and institutional IT administrators, whose insights are vital for understanding the institutional barriers and requirements for AIGC deployment. Furthermore, as our investigation centered on identifying and categorizing user demands, the next logical step involves translating these findings into practice. Future work should therefore concentrate on designing and validating specific technical architectures and prototypes that address these needs, particularly in tackling complex challenges like source verifiability and mitigating AI “hallucinations.” By expanding the scope of inquiry and moving from demand analysis to solution design, the academic community can collectively steer the development of AIGC toward a more responsible, effective, and transformative future.

## Supporting information

S1 File Description: This spreadsheet contains the processed, anonymized minimal dataset required to validate the quantitative analyses presented in this manuscript. It comprises three sheets: 1. Demographics (N = 412): Summary of the survey participants’ disciplines and academic positions. 2. Kano_Analysis: The Better coefficient, Worse coefficient, and Kano category classification for each of the 15 identified AIGC service demands. 3. Cross_Group_Comparison: Aggregated data (means, standard deviations, group sizes) used for the t-tests presented in Table 5 to explore differences across disciplines and career stages.
(XLSX)

## References

[1] Li C, Zheng Z, Du X, Ma X, Wang Z, Li X. KnowBug: enhancing Large language models with bug report knowledge for deep learning framework bug prediction. Knowl-Based Syst. 2024;305:112588. doi: 10.1016/j.knosys.2024.112588

[2] Gallifant J, Fiske A, Strekalova YA, et al. Peer review of GPT-4 technical report and systems card. PLOS Digital Health. 2024;3(1):e0000417. https://doiorg/10.1371/journal.pdig.0000417 10.1371/journal.pdig.0000417 PMC10795998 38236824

[3] Kung TH, Cheatham M, Medenilla A, Sillos C, De Leon L, Elepaño C, et al. Performance of ChatGPT on USMLE: potential for AI-assisted medical education using large language models. PLOS Digit Health. 2023;2(2):e0000198. doi: 10.1371/journal.pdig.0000198 36812645 PMC9931230

[4] de la Torre-López J, Ramírez A, Romero JR. Artificial intelligence to automate the systematic review of scientific literature. Computing. 2023;105(10):2171–94. doi: 10.1007/s00607-023-01181-x

[5] Liu JQJ, Hui KTK, Al Zoubi F, Zhou ZZX, Samartzis D, Yu CCH, et al. The great detectives: humans versus AI detectors in catching large language model-generated medical writing. Int J Educ Integr. 2024;20(1). doi: 10.1007/s40979-024-00155-6

[6] Kosar T, Ostojić D, Liu YD, Mernik M. Computer science education in ChatGPT era: experiences from an experiment in a programming course for novice programmers. Mathematics. 2024;12(5):629. doi: 10.3390/math12050629

[7] Dwivedi YK, Kshetri N, Hughes L. Opinion Paper: “So what if ChatGPT wrote it?” Multidisciplinary perspectives on opportunities, challenges and implications of generative conversational AI for research, practice and policy. Int J Inf Manage. 2023;71:102642. doi: 10.1016/j.ijinfomgt.2023.102642

[8] Albekairi M, Kaaniche K, Abbas G, Mercorelli P, Alanazi MD, Almadhor A. Advanced neural classifier-based effective human assistance robots using comparable interactive input assessment technique. Mathematics. 2024;12(16):2500. doi: 10.3390/math12162500

[9] Rao P, Kumar S, Lim WM, Rao AA. The ecosystem of research tools for scholarly communication. Lib His Technol. 2022;42(4):1132–51. doi: 10.1108/lht-05-2022-0259

[10] Kano N, Seraku N, Takahashi F, Tsuji SI. Attractive quality and must-be quality. J Japanese Soc Qual Control. 1984;14(2):39–48. doi: 10.20684/quality.14.2_147

[11] Slevitch L. Kano model categorization methods: typology and systematic critical overview for hospitality and tourism academics and practitioners. J Hosp Tour Res. 2024;49(3):449–79. doi: 10.1177/10963480241230957

[12] Brynjolfsson E. The Turing Trap: the promise & peril of human-like artificial intelligence. Daedalus. 2022;151(2):272–87. doi: 10.1162/daed_a_01915

[13] Raman R, Pattnaik D, Hughes L, Nedungadi P. Unveiling the dynamics of AI applications: A review of reviews using scientometrics and BERTopic modeling. J Innov Knowl. 2024;9(3):100517. doi: 10.1016/j.jik.2024.100517

[14] Xu R, Sun Y, Ren M, Guo S, Pan R, Lin H, et al. AI for social science and social science of AI: A survey. Inf Process Manag. 2024;61(3):103665. doi: 10.1016/j.ipm.2024.103665

[15] Van Noorden R, Perkel JM. AI and science: what 1,600 researchers think. Nature. 2023;621(7980):672–5. doi: 10.1038/d41586-023-02980-0 37758894

[16] Nyholm S. Artificial intelligence and human enhancement: can ai technologies make us more (Artificially) Intelligent? Camb Q Healthc Ethics. 2023;33(1):76–88. doi: 10.1017/s0963180123000464 37646146

[17] Zhai X. ChatGPT for next generation science learning. XRDS Crossroads ACM Magaz Stud. 2023;29(3):42–6. doi: 10.1145/3589649

[18] Biswas SS. Role of Chat GPT in Public Health. Ann Biomed Eng. 2023;51(5):868–9. doi: 10.1007/s10439-023-03172-7 36920578

[19] Alkire L, Bilgihan A, Bui M (Myla), Buoye AJ, Dogan S, Kim S. RAISE: leveraging responsible AI for service excellence. J Serv Manag. 2024;35(4):490–511. doi: 10.1108/josm-11-2023-0448

[20] Fütterer T, Fischer C, Alekseeva A, Chen X, Tate T, Warschauer M, et al. ChatGPT in education: global reactions to AI innovations. Sci Rep. 2023;13(1):15310. doi: 10.1038/s41598-023-42227-6 37714915 PMC10504368

[21] Liu K, Yao J, Tao D, Yang T. Influence of individual-technology-task-environment fit on university student online learning performance: the mediating role of behavioral, emotional, and cognitive engagement. Educ Inf Technol (Dordr). 2023;:1–20. doi: 10.1007/s10639-023-11833-2 37361766 PMC10157568

[22] Liang CA, Munson SA, Kientz JA. Embracing four tensions in human-computer interaction research with marginalized people. ACM Trans Comput-Hum Interact. 2021;28(2):1–47. doi: 10.1145/3443686

[23] Phillips E, Ososky S, Grove J, Jentsch F. From tools to teammates: toward the development of appropriate mental models for intelligent robots. Proc Hum Factors Ergonom Soc Annu Meeting. 2011;55(1):1491–5. doi: 10.1177/1071181311551310

[24] Alshammari SH, Babu E. The mediating role of satisfaction in the relationship between perceived usefulness, perceived ease of use and students’ behavioural intention to use ChatGPT. Sci Rep. 2025;15(1):7169. doi: 10.1038/s41598-025-91634-4 40021737 PMC11871239

[25] Venkatesh V, Morris MG, Davis GB, Davis FD. User acceptance of information technology: toward a unified view1. MIS Quarterly. 2003;27(3):425–78. doi: 10.2307/30036540

[26] Strzelecki A. Students’ acceptance of ChatGPT in higher education: an extended unified theory of acceptance and use of technology. Innov High Educ. 2023;49(2):223–45. doi: 10.1007/s10755-023-09686-1

[27] Bhaskar P, Misra P, Chopra G. Shall I use ChatGPT? A study on perceived trust and perceived risk towards ChatGPT usage by teachers at higher education institutions. Int J Inf Learn Technol. 2024;41(4):428–47. doi: 10.1108/ijilt-11-2023-0220

[28] Witell L, Löfgren M, Dahlgaard JJ. Theory of attractive quality and the Kano methodology – the past, the present, and the future. Total Qual Manag Bus Excellence. 2013;24(11–12):1241–52. doi: 10.1080/14783363.2013.791117

[29] Weinhardt C, Fegert J, Hinz O, Van Der Aalst WMP. Digital Democracy: a Wake-Up Call. Bus Inf Syst Eng. 2024;66(2):127–34. doi: 10.1007/s12599-024-00862-x

[30] Sarala RM, Post C, Doh J, Muzio D. Advancing research on the future of work in the age of artificial intelligence (AI). J Management Studies. 2025;62(5):1863–84. doi: 10.1111/joms.13195

[31] Sparrow B, Liu J, Wegner DM. Google effects on memory: cognitive consequences of having information at our fingertips. Science. 2011;333(6043):776–8. doi: 10.1126/science.1207745 21764755

[32] Silva TP, Ocampo TSC, Alencar-Palha C, Oliveira-Santos C, Takeshita WM, Oliveira ML. ChatGPT: a tool for scientific writing or a threat to integrity? Br J Radiol. 2023;96(1152):20230430. doi: 10.1259/bjr.20230430 37750843 PMC10646664

[33] Amritesh N, Misra SC, Chatterjee J. Quality framework for credence-based informational services: applying Kano’s method. Total Qual Manag Bus Excellence. 2016;29(1–2):116–47. doi: 10.1080/14783363.2016.1171704

[34] Tenner E. The design of everyday things by donald norman (review). Tech Cult. 2015;56(3):785–7. doi: 10.1353/tech.2015.0104

[35] Taheri B, Okumus F. Conducting mixed methods research. Int J Contemp Hosp Manage. 2023;36(3):995–1004. doi: 10.1108/ijchm-08-2023-1309

[36] Liu Y, Wan F. Unveiling temporal and spatial research trends in precision agriculture: A BERTopic text mining approach. Heliyon. 2024;10(17):e36808. doi: 10.1016/j.heliyon.2024.e36808 PMC11401027 39281636

[37] Xu Q, Jiao RJ, Yang X, Helander M, Khalid HM, Opperud A. An analytical Kano model for customer need analysis. Design Stud. 2009;30(1):87–110. doi: 10.1016/j.destud.2008.07.001

[38] Dwivedi YK, Hughes L, Ismagilova E. Artificial Intelligence (AI): Multidisciplinary perspectives on emerging challenges, opportunities, and agenda for research, practice and policy. Int J Inf Manage. 2019;57:101994. doi: 10.1016/j.ijinfomgt.2019.08.002

[39] Matzler K, Hinterhuber HH. How to make product development projects more successful by integrating Kano’s model of customer satisfaction into quality function deployment. Technovation. 1998;18(1):25–38. doi: 10.1016/s0166-4972(97)00072-2

[40] Papagiannidis E, Mikalef P, Conboy K. Responsible artificial intelligence governance: A review and research framework. J Strat Inf Syst. 2025;34(2):101885. doi: 10.1016/j.jsis.2024.101885

[41] Jobin A, Ienca M, Vayena E. The global landscape of AI ethics guidelines. Nat Mach Intell. 2019;1(9):389–99. doi: 10.1038/s42256-019-0088-2

[42] Crawford K. Atlas of AI: power, politics, and the planetary costs of artificial intelligence. Perspect Sci Christ Faith. 2022;74(1):61–2. doi: 10.56315/pscf3-22crawford

[43] Barredo Arrieta A, Díaz-Rodríguez N, Del Ser J, Bennetot A, Tabik S, Barbado A, et al. Explainable Artificial Intelligence (XAI): Concepts, taxonomies, opportunities and challenges toward responsible AI. Inf Fusion. 2020;58:82–115. doi: 10.1016/j.inffus.2019.12.012

[44] Riley BK, Dixon A. Emotional and cognitive trust in artificial intelligence: a framework for identifying research opportunities. Curr Opin Psychol. 2024;(58):101833. doi: 10.1016/j.copsyc.2024.101833 38991423

[45] Labkoff S, Oladimeji B, Kannry J, Solomonides A, Leftwich R, Koski E, et al. Toward a responsible future: recommendations for AI-enabled clinical decision support. J Am Med Inform Assoc. 2024;31(11):2730–9. doi: 10.1093/jamia/ocae209 39325508 PMC11491642

